# Report on the Changes Which Take Place in the Blood in the Continued Forms of Fever

**Published:** 1857-09

**Authors:** N. S. Davis

**Affiliations:** Chicago, Ill.


THE NORTH-WESTERN

MEDICAL AND SURGICAL JOURNAL.

(NEW SERIES.)

VOL. VI. SEPTEMBER, 1857. NO. 9.

### ORIGINAL COMMUNICATIONS.

ARTICLE I.—*Report on the Changes which take place in the Blood in the Continued Forms of Fever.* Read to the Illinois State Medical Society, June, 1857. By N. S. Davis, M. D., Chicago, Ill.

There are but few subjects of greater importance which could engage our attention than those connected with the pathology of fevers. The doctrines of the ancient humoralists have long since sunk to oblivion, and the dogmas of an exclusive Solidism, as advocated by Darwin, Brown, and Cullen, are rapidly passing in the same direction; while the investigations of the present day are carried on in relation to both solids and fluids, for the sole purpose of developing the facts of physiology and pathology, regardless of all theory; and, though great progress has been made, and much valuable knowledge gained concerning the composition of the blood, the secretions, and some of the organized tissues, by the researches of such men as Andral, Simon, Bernard, Hall, Brown-Sequard, etc., yet more remains to be done, for we can hardly take up any question connected with disease and its progress without finding points yet entirely unelucidated, or explained only by hypothesis.

This is eminently true of the special subject which has been assigned to me for investigation. Concerning the changes which take place in the blood of patients laboring under continued fever, we find but few facts recorded in our medical literature which have been developed by carefully executed experiments and analyses.

Of general statements we have abundance. Thus, Chomel examined the blood in the heart and aorta of thirty fatal cases of typhoid fever, and found "small scanty fibrinous concretions in six, dark coagula in nine, and dark fluid blood in fifteen." Dr. Gerhard describes the blood found in the heart and larger vessels after death from *typhus* fever as uncoagulated, thick in consistence and very dark color. Dr. Jenner says: "The fluid condition of the blood generally was observed in about equal proportions in the subjects dead from typhoid and typhus fevers; but it was far more profoundly diseased, *i. e.*, it deviated far more from its healthy condition in the cases of typhus than in those of typhoid fever." Dr. Upham noted particularly the condition of the blood contained in the heart and larger vessels, after death, from "maculated typhus or ship fever," and he uniformly describes it as "thin, dark and oily," or "fluid, dark and sizy; its clot soft, and readily broken down." Rokitansky says: "The typhus-crasis is marked by the destruction—the diminution of the fibrin, and the comparative preponderence of the blood-globules. The typhus blood is in various degrees fluid, and of a deep purple color. It forms, if any, but scanty, loose, soft, humid, deliquescent coagula, reddened by the imbibition of pigment holding plasma."

Andral and Gavarret, of Paris, made several analyses of the blood in typhoid fever patients, and generally found the fibrin diminished in proportion to the severity of the disease. In no case did they find this constituent increased. Drs. Jones and Sieveking, in their recent work on Pathological Anatomy, state that in several cases of intestinal typhoid fever the globules of the blood had been found increased to 142, and in one to 149 parts in 1,000. My own examinations of the blood in continued fever have chiefly been made during the life of the patient; and in no well marked case have I found it presenting the same appearances as in health.

The two following cases will better illustrate the changes actually presented than any general statement of average results:

CASE I.

*Dec.* 9, 1852.—Mr. Gibbs, a German, aged thirty years, was admitted into the Mercy Hospital in the advanced stage of enteric or typhoid fever. It is now three weeks since the attack commenced, or since he was confined to his bed. He is moderately emaciated; his countenance dull and inexpressive; his face and lips deep red; eyes heavy; mouth dry, with some brown sordes around the teeth; tongue red, pointed, smooth and dry, dullness of hearing; moderate delirium, especially during the night; skin dry, harsh, and above the natural temperature; pulse 112 per minute, soft, and easily compressed; abdomen covered with patches of a red papular eruption, full and tympanitic; urine scanty; discharges from the bowels average three or four per day, are thin, brown and offensive, and during the last eighteen hours they have been more frequent, copious, and tinged with blood. I opened a vein, and took from his arm 1,634 grs. of blood for examination.

TREATMENT.

The patient was ordered a blister over the abdomen, and an emulsion, consisting of oil of turpentine, ʒij.; tinct. opii, ʒij.; gum arabic and white sugar, each ℥ij.; rubbed thoroughly together; with the addition of water, ℥ii.; of which a fluid drachm was to be given every three hours, with beef tea, well salted, for nourishment, and sponging of the surface with warm water every afternoon.

This treatment was continued, without alteration, four days, with a gradual improvement of all the symptoms. Three grains of quinine were then added to each morning dose of the emulsion; and in four days more convalescence was fully established. Soon after convalescence, an abscess of moderate size appeared on one arm, was opened, and discharged considerable unhealthy pus, but soon healed. December 30th, he was discharged from the hospital quite well.

EXAMINATION OF THE BLOOD.

The blood, as it flowed from the vein, was of a very dark color, and, examined under a magnifying power of 850 diameters, soon after it was drawn, the red corpuscles were seen much more variable in size and more irregular in appearance than in healthy blood; one-third of the whole number brought into view were much below the ordinary size, while a few appeared nearly as large as the white corpuscles; and the same disparity in size was maintained after the addition of distilled water. A much larger number were also irregular, shrivelled or plaited than in healthy blood. No white corpuscles were seen on any of the fields examined, though they were diligently sought for. The blood coagulated in the vessel very slowly, and in partially disconnected masses, which, however, slowly united into a uniform clot, preserving the shape of the cup, without any buff, and less firm and compact than the clots of healthy blood.

After standing thirty-six hours, the 1,634 grs. of blood gave 675 grs. of clear serum, and 959 grs. of clot. The several proximate constituents having been carefully separated, presented the following results, viz.:

WHOLE AMOUNT OF BLOOD ANALYSED, 1634 GRAINS.

| | | |
|---|---|---|
| Water, . . . . . | 1327.50 | grains. |
| Red corpuscles, . . | 174.47 | " |
| Albumen, . . . . . | 109.93 | " |
| Fibrin, . . . . . | 4.50 | " |
| Matter soluble in ether, . | 1.10 | " |
| Ash or saline matter, . | 16.50 | " |
| Total, . . | 1634.00 | " |

PROPORTION IN 1000 PARTS.

| | | |
|---|---|---|
| Water, . . . . . | 812.42 | parts. |
| Red corpuscles, . . . | 106.77 | " |
| Albumen, . . . . . | 67.27 | " |
| Fibrin, . . . . . | 2.75 | " |
| Fatty matter, . . . | 67 | " |
| Saline, . . . . . | 10.09 | " |
| Total, . . . . | 999.97 | " |

CASE 2.

Mr. M., native of Ireland, aged 22 years; in the Medical Ward of the Mercy Hospital, Feb. 13th, 1857. Has been sick

18 days with continued fever. Says he took salts and senna in the beginning, which operated excessively, producing numerous large and watery evacuations from the bowels. At present he presents all the characteristics of profound typhus; such as constant muttering delirium, sub-sultus tendinum, brown sordes on the lips and teeth, dry and brown tongue, pulse 120 per minute and weak, abdomen moderately tympanitic and covered with a small red eruption, which extended also over the chest; evacuations from the bowels frequent, dark brown color and very thin. I opened a vein and took 1060 grains of blood for microscopic examination and chemical analysis. The blood drawn was of a dark or purplish color; coagulated slowly and with very little contraction of the clot; while under the microscope the red corpuscles appeared less uniform in size, and less disposed to adhere to each other in the form of *roleau*, than in health. The number of white corpuscles, too, were less than normal. A careful chemical analysis was then made, with the following result, viz.:

WHOLE AMOUNT OF BLOOD ANALYZED, 1060 GRAINS

| | | |
|---|---|---|
| Water, . . . . . . | 838.5 | grains. |
| Red corpuscles, . . . | 138.0 | " |
| Albumen, . . . . . | 71.0 | " |
| Fibrin, . . . . . | 2.0 | " |
| Saline and extractive, . . | 10.5 | " |
| Total, . . . | 1060.0 | " |

PROPORTION IN 1000 PARTS OF BLOOD.

| | | |
|---|---|---|
| Water, . . . . . . | 791.0 | parts. |
| Red corpuscles, . . . | 130.1 | " |
| Albumen, . . . . . | 67.0 | " |
| Fibrin, . . . . . | 1.9 | " |
| Extractive and salts, . . | 10.0 | " |
| Total, . . . | 1000.0 | " |

The two foregoing cases have been selected and the phenomena in connection with the analysis given in detail, because I supposed them to be fair representations of the more grave forms of typhoid and typhus fevers.

I have taken care to preserve the history of all the cases from which blood was analyzed, thereby avoiding a defect which exists in relation to nearly all the analytical tables contained in our works on Pathology.

It will be observed that in neither of the cases detailed was there any great deviation from the normal proportion in any of the proximate elements of the blood. In the first, the water is moderately in excess; the red corpuscles and albumen in the same ratio diminished, while the fibrin and other constituents remained normal. In the second case the water was in normal proportion, the red corpuscles slightly in excess, while both the albumen and fibrin are diminished. From all the analyses I have made, of blood from patients laboring under all the forms of continued fever, I have been led to the following conclusions, viz.:

1st. Taking the average results of twelve analyses of blood from as many different patients with continued fever, I find the relative proportion of the different constituents or proximate elements very nearly normal.

2d. Taking separate cases, I find the blood taken from such as have been accompanied by more than the ordinary amount of watery intestinal discharges, to exhibit an excessive proportion of red corpuscles, while the reverse is true of such as have little or no such excess of intestinal evacuations; and hence that every analysis, to be valuable, must be accompanied by a detailed history of the case.

3d. While we thus find comparatively little change in the amount or relative proportion of the several proximate elements of the blood in continued fever, the alterations in *quality* are striking and uniform. Thus, in every case examined, the color was darker and more purple than normal; the fibrin coagulated very slowly, contracted but little, and the fibrills formed by the coagulation possessed less tenacity than in healthy blood; while the red corpuscles, viewed under the microscope, appear less bi-concave, less uniform in size, and exhibit less attraction or affinity for each other; and the white corpuscles appeared to be very deficient in number.

These are very important changes, and they will not fail to suggest, to the reflecting physician, explanations of many of the more important phenomena and results of continued fever. But as the object of the committee, in making this report, is solely to develope facts, their practical application will be passed by on this occasion, more especially as any attempt of this kind would involve a full consideration of all the changes, functional and structural, which occur in the solids as well as the fluids of the body, and also the consecutive order of their development. Although the duties of this committee were limited to investigations concerning the blood of continued fever patients, yet, for purposes of comparison and contrast, I shall introduce here two cases of periodical fever, with analyses of the blood. Another motive for making the deviation, is found in the fact that such analyses have been very rarely made. On this point, the late Dr. Drake, in his voluminous work entitled a "Systematic Treatise," etc., makes the following observation, viz.: "I do not know that any experiments have been made on the relative proportion of the proximate elements of the blood, in *our* autumnal fever. Andral and Gavarret, in the hospitals of Paris, made such experiments on the blood of seven patients laboring under intermittents of long standing, and found the mean proportion of fibrin to be *three and a third* of one thousand parts, the normal quantity being three. As many chronic cases of the fever are made such by inflammation of some organ, we may presume that in these cases some were complicated with such inflammations. As to the other proximate elements of the blood, the solid residue of the serum was eighty parts in the thousand, the natural proportion; but the blood corpuscles were, on an average, one hundred and four parts in the thousand, while one hundred and twenty-seven is the normal number. Thus, it appears that protracted intermittents produce impoverishment of the blood—spanemia—the condition present in chlorosis; and this accounts in part for the peculiar hue and puffy visage of old ague patients, who so closely simulate chlorosis in their appearance."—See page 820.

The two cases of periodical fever which I have selected to represent that type of disease, and from which the blood was analyzed with great care, are copied from my note books in detail as follows, viz.:

### CASE 1.

Peter Johnson, a Swede, aged thirty-five years, was admitted into the Illinois General Hospital, November 27th, 1852.

### PRESENT APPEARANCE OR SYMPTOMS.

The countenance is dull and inexpressive; face bloated; skin sallow; lips and tongue pale; eyes clear; tongue and mouth clean and moist; universal œdema of the cellular tissue to such an extent that pressure leaves pits on any part of the body and extremities, the latter being much swollen; abdominal dulness and fluctuation, indicating moderate effusion into the cavity of the peritoneum; hepatic dulness appeared to occupy a less space than natural, leading to suspicion of incipient cirrhosis; certainly no evidence of enlargement of the liver or spleen; thoracic resonance good, and respiratory murmur normal, except feebler than usual; rhythm and sounds of the heart normal, except a plain anæmic bellow's murmur; pulse small, soft, and 80 per minute; bowels regular and fæces natural in appearance; urine scanty and loaded with urate of ammonia, giving it a clouded appearance; appetite impaired; muscular weakness very great; position recumbent and dorsal.

### HISTORY.

The patient has had intermittent fever during the last two months, at least, but cannot learn the exact length of time. He has still regular paroxysms every morning, exhibiting a well marked chill, succeeded by a hot stage, during which the pulse is much excited and the anæmic bellow's murmur of the heart very loud and distinct.

### TREATMENT.

Viewing the case as one of extreme loss of tonicity in the solids, and great impoverishment of the blood, from a protracted continuance of that peculiar disturbance of the vital properties, which constitutes the essential pathology of periodical fever, without any satisfactory evidence of local visceral disease, the plain indications of treatment were, first, to interrupt the paroxysms, and, second, to improve the tonicity of the solids and the composition of the fluids.

To fulfil the first, he was ordered chloride of sodium ʒj., and sulphate of quinine 12 grs., to be divided into four powders, and one given at such intervals that the whole would be taken before the time for the next chill.

*Nov. 28th.*—Had a chill this morning, but much lighter than before. Continue same medicine until four more doses are taken, with animal broth well salted for nourishment.

*Nov. 29th.*—No chill or fever this morning. All other symptoms as at first. Ordered a powder composed of chloride of sodium 10 grs., sulph. quinine 2 grs., carb. of iron 3 grs., to be given four times a day, and continue same diet.

*Nov. 30th.*—No return of chills or fever; otherwise all the symptoms, including the debility, the dropsical effusions, etc., were the same as on the day of admission. I now took from his arm, in a clean glass cup, 1448 grs., Troy weight, of blood for examination and analysis. He was then ordered a powder, consisting of extract of sanguinis or dried beef's blood 20 grs., and sulph. quinine 2 grs., to be taken four times a day, with an infusion of juniper berries and *uva ursi* for a drink. A diet of animal broth, milk and bread was continued. This treatment was steadily persisted in with a pretty uniform improvement in all the symptoms for three weeks, when he was discharged from the hospital without any symptoms of intermittent fever or dropsy, and feeling quite well. I met him again in the street on the 30th of December, when he looked and felt entirely well.

EXAMINATION OF THE BLOOD.

The blood coagulated as quickly as in health; the clot becoming firm, small, floating in a clear serum, and retaining accurately the shape of the vessel containing it, without being either cupped or buffed.

Viewed under the microscope, with an object glass magnifying 850 diameters, the red corpuscles differed in no respect from their appearance in healthy blood, except they were in a slight degree more distended or globular. Occasionally one was seen presenting a shrivelled, irregular, and corrugated appearance, but not more so than is sometimes seen in persons apparently in good health. The white corpuscles were certainly deficient in number, only *one* being seen during an examination of six or eight different fields, and the addition, to some of them, of distilled water and acetic acid for the purpose of rendering them more distinct.

The 1448 grs. of blood, after standing, yielded 1048 grs. of clear serum, and 400 grs. of clot. The latter being carefully washed and the product dried over sulph. acid, under the receiver of an air pump, gave 2 grs. of fibrin.

The 1048 grs. of clear serum, by evaporation and careful drying over the acid in a vacuum, yielded 75 grs. of solid matter, and 973 grs. of water.

The 398 grs. of clot, treated in the same manner, gave 98 grs. of solid matter, and 300 grs. of water.

The 75 grs. of solid matter from the serum, and the 98 grs. from the clot, after digesting twenty-four hours in strong sulphuric ether, yielded to the latter only 1 gr. of soluble matter. The solid matter of the serum left, after incineration in a porcelain crucible, 1.5 grs. of ash. That of the clot yielded, by the same process, 4.5 grs. of ash.

The combined results may be stated in tabular form, as follows, viz.:

WHOLE AMOUNT OF BLOOD EXAMINED, 1448 GRAINS.

| | | |
|---|---|---|
| Water, . . . . . | 1273.0 | grains. |
| Red corpuscles, . . . . | 72.1 | " |
| Albumen, . . . . . | 93.9 | " |
| Fibrin, . . . . . . | 2.0 | " |
| Matter soluble in ether, . | 1.0 | " |
| Ash or saline matter, . . | 6.0 | " |
| Total, . . . . | 1448.0 | " |

PROPORTION IN 1000 PARTS OF BLOOD.

| | | |
|---|---|---|
| Water, . . . . . | 879.14 | parts. |
| Red corpuscles, . . . . | 49.79 | " |
| Albumen, . . . . . | 64.84 | " |
| Fibrin, . . . . . . | 1.38 | " |
| Matter soluble in ether, . | .69 | " |
| Saline matter, . . . . | 4.14 | " |
| Total, . . . . | 999.98 | " |

CASE 2.

The description of this case may be condensed from my note book, as follows, viz.:

Mr. Sampson, a Swede, aged 23 years, was admitted into the hospital, Nov. 28, 1852. Says he has had a quotidian intermittent almost constantly during the last six or eight weeks. At present he is moderately emaciated; countenance sallow; lips and tongue pale; pulse 85 per minute, and easily compressed; cutaneous and urinary secretions diminished, with slight tendency to diarrhœa, and great muscular weakness. No signs of œdema or of visceral disease, either in the chest or abdomen.

His intermittent paroxysms were promptly arrested by the exhibition of chloride of sodium and sulph. of quinine, in the same manner as in case No. 1. On the fourth day after admission, no chills having recurred since the second day, I took 1410 grs. of blood from his arm for examination, and ordered him extract of sanguinis, 15 grs., four times a day, with a nutritous diet. He continued this treatment steadily, with uniform improvement, and was discharged, apparently well, near the end of the third week after admission.

EXAMINATION OF THE BLOOD.

In coagulating and under the microscope, this blood presented the same phenomena and appearances as No. 1. The clot was larger in proportion to the serum, but there was the same shape, firmness, etc., and the same deficiency of white corpuscles. The different steps of analysis were conducted in all respects the same as in the former case, and the result stated in tabular form is as follows, viz.:

WHOLE AMOUNT OF BLOOD EXAMINED, 1410 GRAINS.

| | | |
|---|---|---|
| Water, . . . . . | 1182.50 | grains. |
| Red corpuscles, . . . | 116.74 | " |
| Albumen, . . . . . | 98.26 | " |
| Fibrin, . . . . . . | 3.50 | " |
| Matter soluble in ether, . | 1.00 | " |
| Ash or saline matter, . . | 8.00 | " |
| Total, . . . | 1410,00 | " |

PROPORTION PER 1000 PARTS.

| | | |
|---|---|---|
| Water, . . . . . | 838.65 | parts. |
| Red corpuscles, . . . . | 82.79 | " |
| Albumen, . . . . . | 69.68 | " |
| Fibrin, . . . . . . | 2.48 | " |
| Fatty matter, . . . . | .71 | " |
| Saline matter, . . . . | 5.67 | " |
| Total, . . . . | 999.98 | " |

The two cases here given in detail are selected as fair representatives of the uncomplicated periodical fever in different stages of its progress.

The first, represents the extreme changes induced in the fluids and solids of the body by the disease when protracted and uninfluenced by medical treatment. The second, represents the changes induced in the fluids in the more recent cases, such as are daily met with in practice. All my analyses of blood from patients laboring under attacks of simple intermittent and remittent fevers, lead to the following conclusions, viz :

1st. The qualities or properties of the several constituents of the blood appear normal.

2nd. The relative quantity of the different proximate elements of the blood undergo marked and important changes. The red corpuscles undergo a marked and nearly uniform diminution in proportion to the duration of the disease, sometimes presenting no more than one-third of the normal proportion. The white corpuscles were also deficient in all the specimens examined by me; while the albumen and fibrin were only slightly below the normal proportion.

3rd. The foregoing changes in the solid and organized constituents of the blood, leave the water relatively in excess, rendering the whole mass of the circulating fluid thinner than normal, and consequently favoring the occurrence of congestions and dropsical effusions.

These conclusions are strikingly diverse from those derived from an examination of the blood during the progress of continued fever, as already given. In one respect they differ from the results obtained by MM. Andral and Gaverret, who found the proportion of fibrin increased in the blood of their patients with intermittent fever. But as Dr. Drake suggests, it is quite probable that the excess of fibrin in their cases was owing to local inflammations as complications, all their cases being chronic or relapsing agues, and consequently not fair representatives of the disease.

Members of the society will also notice that in one respect the results I have obtained from analyses of blood in continued fever, differ from the statements of M. Andral and from the generally received opinions of the profession. I allude to the relative proportion of fibrin, which is stated to be deficient in all the graver cases of typhoid and typhus fevers, but which was found very little below the normal standard in any of the cases examined by me. I am satisfied that the general impression of deficiency of fibrin has been exaggerated from two causes: First, its slow and imperfect coagulation in the blood found in the heart and vessels after death, or even when drawn from a vein during life, gives at once the impression of deficiency of the coagulating material. Second, the method of separating the fibrin adopted by Andral, consisted in stirring or whipping the freshly drawn blood with a bundle of sticks, so as to entangle the fibrin on them. It seems to me obvious that whenever the *quality* of the fibrin is so impaired that it coagulates slowly and its fibres contain little tenacity, this would constitute a very imperfect method of separating it. To avoid loss from the diminished coagulability and tenacity of the fibrin, I adopted in all my analyses the plan of allowing the blood to remain at rest nearly twenty-four hours, thereby giving time for the clot to be fully formed. It was then inclosed in a piece of fine linen cloth and washed with water, until nothimg but the pure white fibrin was left on the cloth; after which, it was thoroughly dried and weighed. By this process the fibrin is more perfectly separated than by simply stirring it as in the other method, especially when this constituent after solidification possesses but little tenacity.

The results of my investigations thus far suggests several questions of much interest and practical importance in relation to the pathology of both types of fever.

1st. What is the immediate cause of such marked impairment of the properties of the several constituents of the blood in continued fever? Is it some poison introduced into the blood from without, or an excess of some one of the gases naturally existing in the blood, which are known to lessen the coagulability of fibrin and give to the whole blood a purplish hue, such as the carbonic and ammoniacal gases? Dr. Richardson, of London, in a recent prize essay, claims that the fibrin of the blood in a healthy state is held in solution while in the living system by the presence of a certain quantity of ammoniacal gas. Is there an excess of this substance present in the blood of continued fever patients?

2d. What are the pathological conditions or peculiar changes in the properties of the solid or organized structures of the body in both types of fever, and what relation do such changes bear to the changes in the blood?

To answer these questions intelligibly requires much and careful investigation, embracing many new and well devised experiments and analyses, which want of time has hitherto prevented me from attempting.

I hope, however, that members of the society will give the subject their careful attention, always remembering that one fact or truth well established is of more value to science than an hundred theories.

